# The Δ133p53 Isoform Reduces Wtp53-induced Stimulation of DNA Pol γ Activity in the Presence and Absence of D4T

**DOI:** 10.14336/AD.2016.0910

**Published:** 2017-04-01

**Authors:** Kai Liu, Yunjin Zang, Xianghua Guo, Feili Wei, Jiming Yin, Lijun Pang, Dexi Chen

**Affiliations:** ^1^Beijing Institute of Hepatology, Beijing You An Hospital, Capital Medical University, Beijing 100069, China; ^2^The Affiliated Hospital of Qingdao University, Organ Transplantation Center, Qingdao, Shandong 266003, China

**Keywords:** mtDNA, mtBER, p53 isoform, stavudine (d4T), mitochondria

## Abstract

The mitochondrial toxicity of nucleoside reverse transcriptase inhibitors (NRTIs) is due to the inhibition of mitochondrial DNA (mtDNA) polymerase γ (pol γ). Previous studies have shown that wild type p53 (wtp53) can interact with pol γ and mtDNA to enhance mitochondrial DNA base excision repair (mtBER) activity and increase the accuracy of DNA synthesis. The N-terminal transactivation domain and central specific DNA-binding domain of p53 play critical roles in the stimulation of BER. In this study, we identified the possible roles of wtp53, Δ40p53 and Δ133p53 in regulating mtDNA pol γ activity in cells with d4T treatment. The results show that Δ40p53 and Δ133p53 can exist in mitochondrial fragments and form polymers with themselves or wtp53. Unlike wtP53, Δ133p53 alone cannot increase DNA pol γ activity. More importantly, we found that Δ133p53 played a negative role in p53 stimulation of DNA pol γ activity when studied in d4T-treated and d4T-untreated mitochondrial extracts. Gel shift data also indicate that Δ40p53 and Δ133p53 cannot interact with APE. Wtp53 and Δ40p53 can act antagonize the effect of d4T inhibition of DNA pol γ activity. However, when wtp53 interacted with Δ133p53, DNA pol γ activity was significantly decreased. Conclusion: Δ133p53 negatively regulates p53’s stimulation of pol γ in the presence and absence of d4T.

In the last twenty years, highly active anti-retroviral therapy (HAART) has made major progress in the treatment of HIV-1 infection [1, 2]. Since the clinical introduction of HAART in 1996, the frequency of the opportunistic infections (OIs), which were once accompanied with a high mortality, have decreased dramatically. Nucleoside reverse transcriptase inhibitors (NRTIs) form the backbone of HAART. Despite the clinical benefits and a significant decline in AIDS related morbidity and mortality, long-term treatment with HAART can be associated with potentially severe adverse effects resulting from mitochondrial toxicity [3-5]. DNA pol γ inhibition has been proposed as the main reason for the mitochondrial toxicity induced by NRTIs [6-8].

D4T was one of the first NRTIs to be recommended as a treatment for AIDS. As a first-line treatment, it significantly reduces the HIV-1 viral load and delays the progression of AIDS. However, due to its severe mitochondrial toxicity, d4T as a first-line therapy was removed from the antiretroviral (ARV) guidelines in the United States in 2006. However, since there are only a limited number of free treatments available in China, d4T is still widely used as a first-line treatment for AIDS patients. Previous studies have shown that the high mitochondrial toxicity of d4T is due to the inhibition of mitochondrial DNA pol γ [9-12]. DNA pol γ is a polymerase unique to the mitochondria and is involved in the replication of mtDNA as well as mtBER [13-15]; the mitochondrial toxicity of d4T is caused by the dysfunction of these processes.

The p53 tumor suppressor protein, often called the ‘guardian of the genome’, is a central mediator of the cellular damage response. The p53 pathway plays a major role in maintaining genomic stability through cell cycle delay accompanied by the repair of DNA damage [16-21]. The evidence indicate that BER induced by the base-damaging agent methyl methanesulfonate (MMS) is partially deficient in cells, which lack functional p53 [22]. This suggests that the activity of BER might also be dependent on p53. Previous studies have shown that wild-type p53 (wtp53) can combine with mtDNA and pol γ to enhance mtBER activity and maintain mitochondrial genetic stability [22-24]. It has been shown that the p53 genotype (p53^+/-^) does not significantly affect the gene expression profile induced by AZT/3TC treatment [25]. Other studies have shown that both AZT and AZT-3TC treatments can induce small but significant increases in the frequency of hypoxanthine-guanine phosphor-ribosyltransferase (Hprt) mutant lymphocytes in p53^+/-^ mice, but not in p53^+/+^ mice [26]. p53 overexpression has also been detected in some AZT-treated mouse neoplasms [6]. However, the role of p53 in NRTI-induced mitochondrial toxicity remains unclear.

It has been shown that the p53 gene can initiate transcription from an alternative promoter located in intron 4 [27] and can be transcribed to produce multiple splice variants. To date, at least nine p53 protein isoforms have been reported [27], some of which can be detected in normal tissue and tumors and it is thought that some isoforms may play a role in tumor pathogenesis. Nuclear BER and mtBER in HCT116 p53^+/+^ cells were confirmed to be stimulated by wtP53 [22, 28]. The N-terminal transactivation domain and central DNA-binding domain of p53 play a critical role in stimulating BER and Δ40p53 has a major influence over p53 activity by controlling p53 ubiquitination and cell localization [29, 30].

In this study, we identified the possible mechanisms of Δ40p53 and Δ133p53 (isoforms of wtp53) regulating mtDNA poly γ activity. We found that the Δ40p53 and Δ133p53 can localize in the mitochondrial fragment and form polymers with wtp53. We examined the effect of wtp53, Δ40p53 and Δ133p53 on mtBER using an *in vitro* nucleotide incorporation assay and report that Δ133p53 alone cannot increase DNA pol γ activity, however, it can reduce the DNA pol γ activity induced by p53 in both d4T-treated and d4T-untreated mitochondria. Gel shift studies indicate that Δ133p53 cannot interact with AP-endonuclease 1 (APE1). These results suggest that Δ133p53 could play a significant positive role in d4T induced mitochondrial toxicity.

## MATERIALS AND METHODS

### Cell culture, d4T treatments, p53 RNAi transfection and reagents

H1299 cells were obtained as a generous gift from C. Lopez (Oregon Health Science University). H1299-Δ133p53 and H1299-Δ40p53 stable cell lines were established in our lab. The A549 wtp53 cell line was a gift from Dr. X, He (Beijing University). Cells were cultured in DMEM medium supplemented with 10% heat-treated fetal bovine serum (FBS), 290 µg of L-glutamine, 100U penicillin and 100 µg of streptomycin per ml and maintained in logarithmic growth at 37°C in 5% CO_2_. The d4T was received as a gift from the Dongbei Pharmacy Company of China. Recombinant p53, Δ133p53, Δ40p53 and APE1 were generated by cloning cDNAs into a His-tagged expression vector (Invitrogen, Carlsbad, CA) following the manufacturer’s instructions. After Isopropyl-L-thio-β-D-galactopyranoside induction in BL21 bacteria, expressed proteins were purified on Ni-NTA agarose according to the manufacturer’s protocol (Qiagen, Valencia, CA) then stored at -80°C. Purified protein was quantified using silver-stained SDS-PAGE with bovine serum albumin standards. Klenow fragment and T4 DNA ligase were obtained from Invitrogen. For adenovirus infections, a p53-expressing adenovirus and a control (empty vector) were produced using the AdEasy adenoviral vector system (Stratagene, USA); adenovirus infections were carried out using standard techniques with a multiplicity of infectivity (MOI) of 1×10^10^. The pcDNA™ 6.2-GM-p53-RNAi vector was cloned using a pcDNA™ 6.2-GM RNAi kit according to the manufacturer’s instructions (Invitrogen, Shanghai China). The oligonucleotide used were as follows: p53-RNAi40-s1: 5’-TGCTGTACGTGCAAGTCACAGACTTGGTTT TGGCCACTGACTGACCAAGTCTGTCTTGCACGTA-3’; p53-RNAi40-As1: 5’-CCTGTACGTGCAAGACA GACTTGGTCAGTCAGTGGCCAAAACCAAGTCTGTGACTTGCACGTAC-3’. Cloned oligonucleotides were sequenced to confirm that no mutations were introduced during the cloning processes. Gene transfections were carried out in 6-well plates and 2×10^5^ cells/well (Δ40p53-H1299, A549) were seeded using 200 of each oligonucleotide per well and transfected in triplicates using the Fugene6 Transfection Reagent.

### Western blotting and immunoprecipitation

Immunoprecipitation and immunoblotting experiments were carried out using standard chemiluminescence procedures as described previously [31]. Polyclonal p53, HSP60 and tubulin antibodies were purchased from Santa Cruz (CA, USA). Pol γ antibodies were purchased from Abcam (Beijing, China) and APE1 was a gift from Dr. G. Fraspi (Oregon Health Science University). HRP-conjugated secondary antibodies were purchased from the Jackson Laboratory.

### DNA isolation and quantitative real-time PCR

Total DNA was isolated using DNA isolation kits (Qiagen, Beijing China) according to the manufacturer’s instructions. Quantitative PCR (qPCR) assays were performed as previously described with minor modifications [32]. The TaqMan 7900HT system was used to perform real-time PCR amplification of the mtDNA region CoxII (Forward Primer: 5′- ccccacattaggcttaaaaacagat -3′, Reverse Primer: 5′- tatacccccggtcgtgtagcggt-3′, Probe: 5' FAM -caattcccggacgtctaaaccaaaccactttc- TAMRA-3') and β-actin (Forward Primer: gccatcctgcgtctggacctggct, Reverse Primer: tgatgacctggccgtcaggcagctc, Probe: 5'-FAM-gccgggacctgactgactacctcatga- TAMRA-3'). All primers and probes were obtained from Invitrogen (Shanghai, China). Real-time PCR reactions were performed in triplicates for each gene. Data analysis was performed using Microsoft Excel.

### Preparation of purified mitochondria and qPCR assay for DNA polymerase γ activity

All procedures were carried out at 4°C. Mitochondria were isolated using differential centrifugation and Percoll gradient centrifugation adapted from methods previously described [33]. The purified mitochondrial pellet (total 5 µg) was resuspended in 20 µl of DNA pol γ assay solution (0.1 pmol template A and B, 20 µg poly dI/dC, 45 mM HEPES-KOH (pH 7.8), 70 mM KCl, 7.4 mM MgCl_2_, 0.9 mM DTT, 0.4 mM EDTA, 40 mM phosphocreatine, 2.5 μg creatine phosphokinase, 20 μg/ml BSA, 3.4% glycerol, 2 mM ATP, 20 μM dNTP and 5 U T4 DNA ligase) at 30°C for 45 minutes. The template (from the *in vitro* DNA pol γ activity assay) was used at 50 fmol (1:1000 elution). qPCR was performed using an annealing temperature of 65°C for 40 cycles with probe 1 (Fam) plus probe 2 (Tet).

### In vitro ^32^P-dGTP incorporation assay for DNA polymerase γ activity

The ^32^P-dGTP incorporation assay was performed as previously described with minor modifications [34]. Briefly, equivalent amounts of mitochondrial extracts prepared from H1299 cells were incubated with AP-containing (opposite a C) double-stranded oligonucleotide BER templates (Sigma, Beijing China). Reactions were carried out at 32°C in a 50 μl reaction volume in buffer containing 45 mM Hepes-KOH (pH 7.8), 70 mM KCl, 7.4 mM MgCl_2_, 0.9 mM DTT, 0.4 mM EDTA, 40 mM phosphocreatine, 2.5 μg creatine phosphokinase, 20 μg/ml BSA, 3.4% glycerol, 2 mM ATP, 20 μM each of dATP, dTTP and dCTP, 8 μM dGTP, 2 μCi of 32P-dGTP, 2 μl APE1 and 10U T4 DNA ligase. Reactions were terminated by the addition of 240 μg/ml proteinase K, 1% SDS and 20 mM EDTA, then incubating for 30 min at 37°C. Reaction products were resolved on 15% polyacrylamide gels containing 7 M urea, followed by autoradiography and quantification by scanning densitometry.

### Electrophoretic Mobility Shift Assays (EMSAs)

EMSA assays were performed as previously described [29]. For a typical reaction (20 μl), 0.05 pmol 5’-end ^32^P labeled AP-DNA oligos were mixed with APE1, Mt-extract (10ug) and either p53, Δ40p53, Δ133p53, or d4T in binding buffer (50 mM HEPES, pH 7.5, 100 mM KCl, 10 mM MgCl_2_, 0.1 mM EDTA, 40 μg poly dI/dC and 10% glycerol). The mixture was incubated on ice for 20 minutes and protein-DNA complexes were resolved on 8% polyacrylamide gels in 0.5X TBE followed by autoradiography.

### Oligomerization assays

Oligomerization assays were performed as previously described [35]. Briefly, H1299, Δ40p53-H1299, or Δ133p53-H1299 cells were infected with rAD-p53. Mitochondrial lysates were prepared as described above for Western blotting. For standardization of transfection efficiency, equal amounts of protein were used, which were treated with 0, 0.01 or 0.1% glutaraldehyde for 5 min on ice. Following addition of SDS sample buffer, samples were resolved by SDS-PAGE on a 4-20% gradient gel. Western blot analysis was performed using polyclonal antibodies (Santa Cruz, CA, USA), to detect wtp53, Δ40p53-H1299 and Δ133p53.

### Primers sequences (A, B, C, D and E) used in in vitro BER

Primer A: 5’-ATGGCGGGGCTCTCCAGAACATCAT CAATTCCCGGACGTCTAAACCAAACCACTTTCA-3’; Primer B: 5’-AGGTCCAGGTCTGGAAGGCTGTG GGCAAGGTCATAT-3’; Primer C: 3’-(ddG)CCTGCAG ATTTGGTTTGGTGAAAGTGTCCAGGTCCAGACC-5’; Primer D: 5’ATGGCGGGGCTCTCCAGAACATCA TCCGGGACCTGACTGACTACCTCATGAACAGGTCCAGGTCTGGAAGGCTGTGGGCAAGGTCATAT-3‘; Primer E: 3’-(ddG)TAGGCCCTGGACTGACTGATGG AGTACTTGTCCAGGTCCAGACC-5’

### Statistical Analysis

All data shown are the results of at least three independent experiments and expressed as the mean ± SD. The differences between groups were compared using Student’s t-test. Differences were considered statistically significant at confidence levels of *p*<0.05, *p*<0.01 and p<0.001 as indicated.

## RESULTS

### Δ40p53 and Δ133p53 can translocate to the mitochondria and form polymers with endogenous wtp53

**Figure 1.**
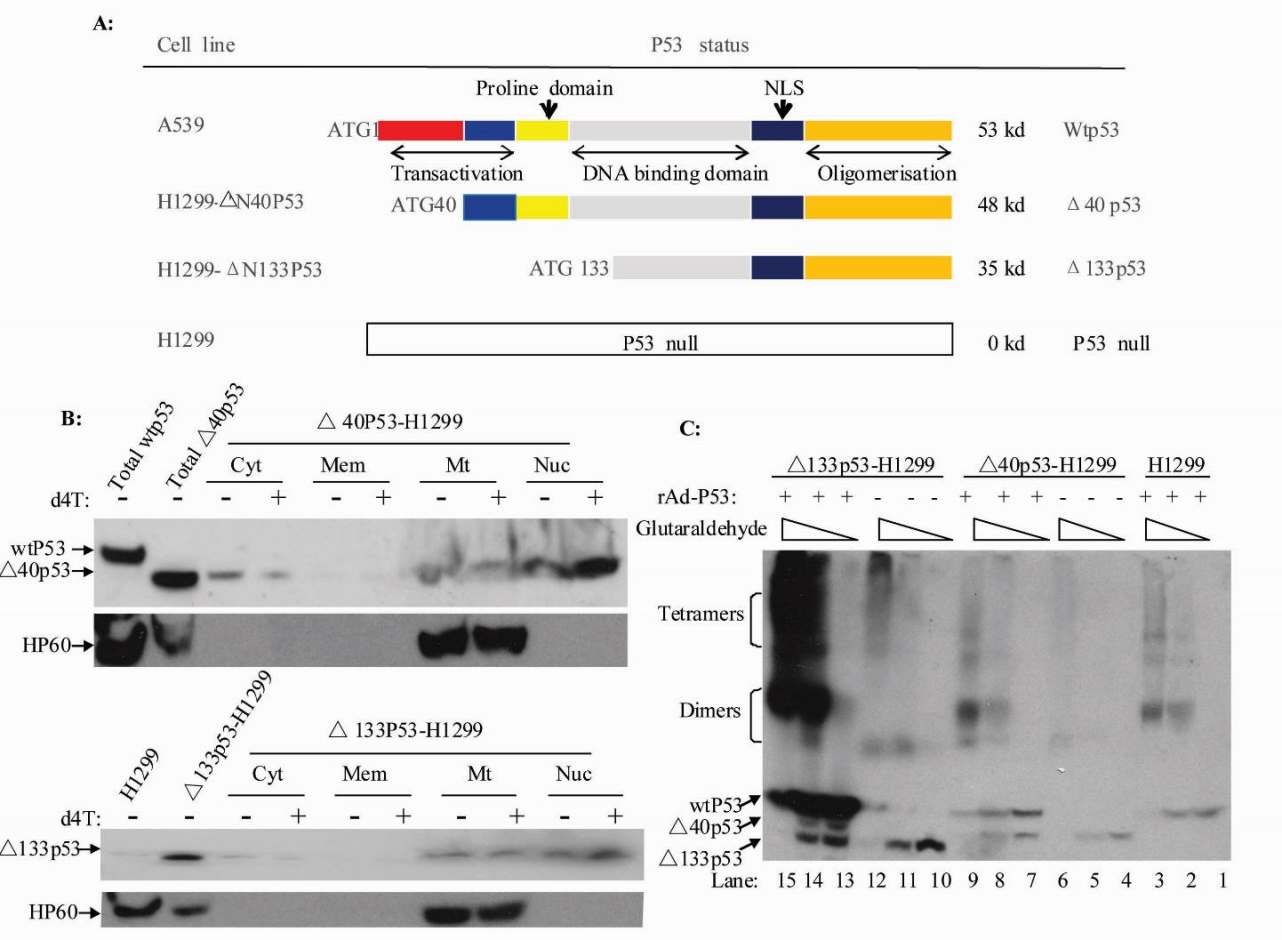
**p53 protein isoforms (Δ40p53 and Δ133p53) located in the mitochondria, alone and as oligomers with p53**. (**A**) Schema of the p53 and p53 isoforms (Δ40p53 and Δ133p53) theoretically encoded by the human p53 gene. (**B**) Immunoblots on crude fractions (cytosol, ER, mitochondria and nuclear) from Δ40p53-H1299 andΔ133p53-H1299 cells treated with the indicated amounts of d4T for 24 hours. (**C**) Oligomerization analysis of Δ40p53 and Δ133p53 with p53.

Previous studies have shown that p53 can affect the stability of mitochondrial DNA. p53 isoforms have recently been discovered in different tumor cell lines and tumor tissues, which may play a role in the development of tumors [27, 36, 37]. However, the role of p53 isoforms in mitochondrial DNA replication and damage repair remains unclear. More than nine p53 isoforms have been reported. In this study, we focused on the α-form of Δ40, Δ133 and wtp53 (Fig. 1A).

Previous studies have shown that wtp53 can enter the mitochondria and become involved in mtBER [22]. In this study, we first examined whether p53 isoforms can enter and form polymers in the mitochondria. We found that both Δ40p53 and Δ133p53 can enter the mitochondria and were not associated with d4T (Fig. 1B). Mitochondrial extracts were treated with different concentrations of glutaraldehyde for 5 min on ice to identify the polymerized forms. The results show that Δ40p53 and Δ133p53 alone or with wtp53 can form dimers and tetramers (Fig. 1C). These results suggest Δ40p53 and Δ133p53 could play a role in the stability of mitochondrial genomic DNA.

### Wtp53 but not Δ40p53 or Δ133p53 can stimulate DNA pol γ activity while d4T attenuates DNA pol γ activity induced by wtp53

**Figure 2.**
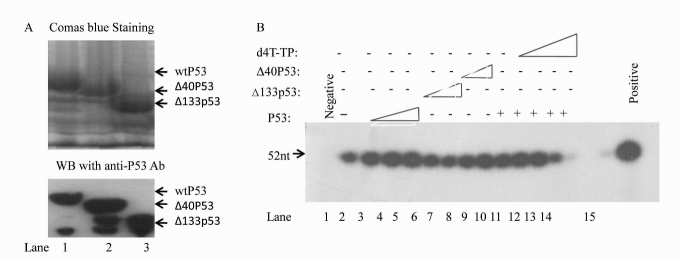
***In vitro* BER assay with purified wtP53, Δ40p53 and Δ133p53 fusion proteins showing that Δ40p53 and Δ133p53 cannot induce mtBER but can attenuate mtBER activity induced by wtp53**. (A) wtP53, Δ40p53 and Δ133p53 His fusion proteins were stained with Coomassie blue (upper panel) and identified by Western blotting with anti-P53 antibodies (lower panel). (B) Purified p53, Δ40p53 and Δ133p53 protein (100, 500 and 1000 ng, lanes 3-9) or d4T (10, 50 and 300 nM, lanes 11-14) were added to BER reaction mixtures containing both whole-mitochondrial extracts obtained from H1299 cells and T4 DNA ligase. The templates were treated with T4 DNA ligase and Klenow fragment was used as a positive control (lane 15).

Whether or not the Δ40p53 and Δ133p53 isoforms of wtp53 can stimulate DNA pol γ activity was unclear. To examine this, we performed *in vitro* DNA pol γ activity assays with mitochondrial extracts from the H1299 cells treated with wtp53, Δ40p53 and Δ133p53 fusion proteins, as well as d4T. We first purified His6-wtp53, His6-Δ40p53 and His6-Δ133p53 fusion proteins from BL21 *E. coli*. The Coomassie blue (the upper panel) and western blotting (the lower panel) results are shown in Figure 2A. We then examined the effect of wtp53, Δ40p53, Δ133p53 on DNA pol γ activity. As in previous reports, we found that wtp53 stimulates mitochondrial DNA pol γ activity (Fig. 2B). However, Δ40p53 only had weak stimulation of DNA pol γ activity while Δ133p53 failed to stimulate DNA pol γ activity even at high concentrations (Fig. 2B, lanes 6-9). mtDNA pol γ activity was significantly attenuated by d4T (Fig. 2B). These results suggest that the N-terminal domain of p53 (40_aa_ and 40_aa_-_132aa_) play an important role in the stimulation of mtDNA pol γ activity and that d4T strongly attenuates the mtDNA pol γ activity induced by wtP53.

**Figure 3.**
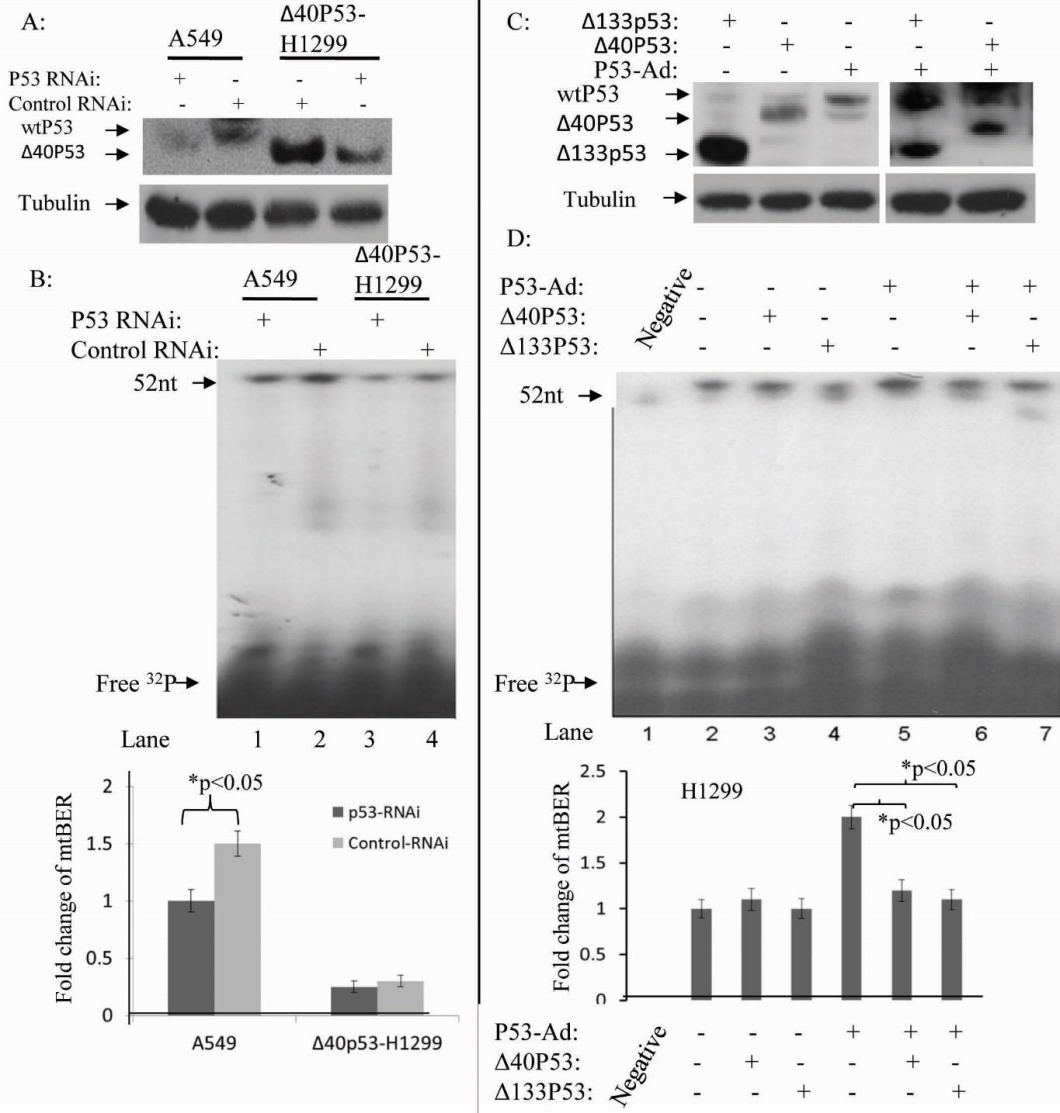
***In vitro* BER assay with mitochondrial extract including endogenous wtP53, Δ40p53 and Δ133p53 indicates that Δ40p53 and Δ133p53 cannot induce mtBER but can attenuate mtBER activity induced by wtp53**. (A) A representative Western blot shows that wtP53 and Δ40p53 expressions were suppressed by p53 RNAi. (B) Total mitochondrial BER activities in A549 cells (lanes 1 and 2) and Δ40p53-H1299 cells (lanes 3 and 4) were decreased through p53 RNAi. Points, means of triplicate experiments; bars, SD; *, P < 0.010 (unpaired two-tailed t test). (C) A representative Western blot showed p53 expression using the different p53 constructs. (D) Δ40p53 and Δ133p53 protein decreased mtBER activity induced by the wtp53 (lanes 6 and 7). Points, means of triplicate experiments; bars, SD; # P < 0.010 (unpaired two-tailed t test).

To examine whether endogenous wtp53 and Δ40p53 can restore mtDNA pol γ activity, we transfected pcDNA™6.2-GM-p53-RNAi into the wtp53 cell line A549 and into H1299-Δ40p53 cells. The level of P53 and Δ40p53 from mitochondrial extracts of the pcDNA™6.2-GM-p53-RNAi treated and untreated A549 and H1299-Δ40p53 cells were detected (Fig. 3A). Western blotting results show that the wtp53 levels in A549 cells and Δ40p53 levels in H1299-Δ40p53 cells were decreased by p53-RNAi. *In vitro* analysis with these mitochondrial extracts showed that the mtDNA pol γ activity in A549 cells was 2-fold higher than in p53 RNAi treated A549 cells (Fig. 3B). However, mtDNA pol γ activity was not significantly different between H1299-Δ40p53 and p53-RNAi treated H1299-Δ40p53 (Fig. 3B). This suggests that Δ40p53 had a weak effect on mtDNA pol γ activity compared to wtP53. Due to lack of suitable siRNAs to inhibit the expression of Δ133p53, we have not performed an analysis of mitochondrial endogenous repair activity in H1299-Δ133p53 cells. In order to study whether endogenous Δ40p53 and Δ133p53 play a dominant-negative role in wtp53 inducing mtDNA pol γ activity, H1299-Δ133p53, H1299-Δ40p53 and H1299 cells were infected by rAD-p53 for 36 hours before extraction of mitochondria for mtDNA pol γ activity analysis. WtP53 co-expression with Δ133p53 and Δ40p53 in H1299-Δ133p53 and H1299-Δ40p53 cells were confirmed (Fig. 3C). We then isolated mitochondrial extracts for DNA pol γ activity assays. The results indicate that Δ133p53 and Δ40p53 can inhibit mtDNA pol γ activity induced by p53 (Fig. 3D). These results suggest that Δ40p53 and Δ133p53 play a dominant negative role in the regulation of p53 stimulation of mtDNA pol γ activity.

**Figure 4.**
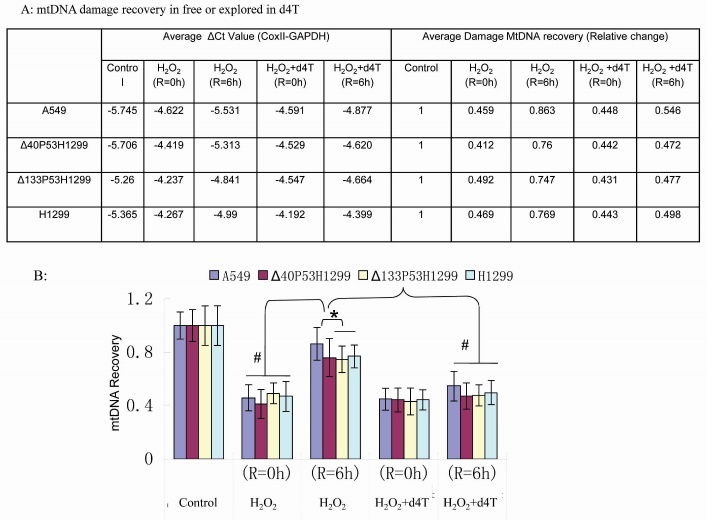
**Endogenous mtDNA damaged by H_2_O_2_ was recovered from cell lines with different p53 constructs**. (**A**) The average ΔCt value (mtDNA vs GAPDH) and the ratio of mtDNA recovery in A549, Δ40p53H1299, Δ133p53H1299 and H1299 cells. (**B**) The corresponding graph shows that mtDNA damage recovery is significantly higher in A549 cells and that mtDNA recovery is strongly attenuated by d4T. The data represent the average of three independent experiments; bars, SD; *P < 0.05 and # *P* < 0.01.

### Damaged mtDNA recovery is significantly different among A549, H1299-Δ40p53, H1299-Δ133p53 and H1299 cells; d4T reduces damaged mtDNA recovery.

Due to the presence of high levels of reactive oxygen species (ROS) in the mitochondrial matrix, mtDNA mutations are frequent in cells and need to be corrected by the mtBER pathway in order to maintain mtDNA stability [38]. P53 plays an important role in this repair process via stimulation of DNA poly γ activity [23]. *In vitro* BER assays have shown that the isoform Δ133p53 significantly attenuates the mtBER activity induced by wtP53 (Fig. 3). In order to identify the role of different p53 isoforms in damaged mtDNA recovery, we performed real-time qPCR to analyze mtDNA integrity and damage repair. Our results indicate that in H1299 cells and H1299-Δ133p53 cells, less mtDNA was recovered compared with A549 cells and H1299-Δ40p53 cells. The mtDNA recovery was not significantly different between H1299 and H1299-Δ133p53 cells, but the mtDNA recovery was significantly different between the A549 and H1299-Δ40p53 cells (Fig. 4A). It is known that d4T reduces the activity of DNA poly γ [39, 40] and in this study, d4T robustly inhibited DNA poly γ activity induced by p53 in the *in vitro* assays (Fig. 2 and 3). We confirmed this finding using qPCR in the *in vivo* assays after cells were treated with both H_2_O_2_ and d4T (Fig. 4B). The results showed that the recovery ratio of mtDNA was 43%, 39%, 32% and 34% in A549, H1299-Δ40p53, H1299-Δ133p53 and H1299 cells after 12 hours of d4T and 1 hour of H_2_O_2_ treatment following 6-hour recovery in H_2_O_2_ free medium (while still containing d4T), respectively.

**Figure 5.**
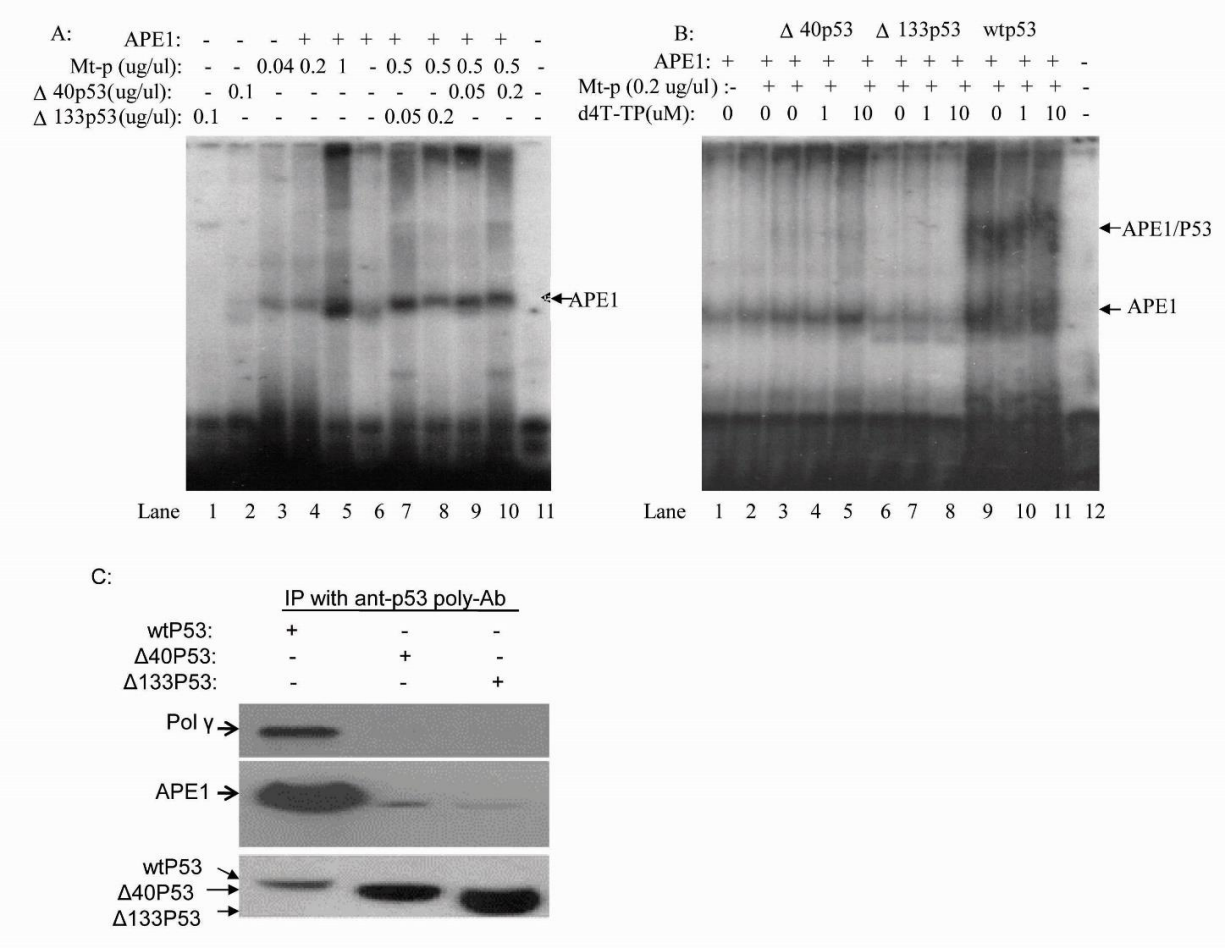
**WtP53 but not Δ40p53 and Δ133p53 stabilizes the interaction between APE and AP-DNA containing whole-mitochondrial extracts**. (**A**) Δ133p53 attenuated APE-interaction with AP-DNA (lanes 7 and 8); and Δ40p53 weakly stimulated APE interaction with AP-DNA (lanes 9 and 10). (**B**) d4T does not affect APE interaction with AP-DNA. p53 can interact with the APE-AP-DNA complex (lanes 9-11) but Δ40p53 and Δ133p53 cannot interact with the APE-AP-DNA complex (lanes 3-8). (**C**) A representative immunoprecipitation and Western blot from different p53 proteins stabilizing the interaction between APE and AP-DNA containing oligos (A and B). The data show that only wtP53 interacts with APE1 or DNA pol-γ.

### Wtp53 but not Δ40p53 or Δ133p53 interacts with the APE-AP oligonucleotide complex and d4T does not affect the interaction between DNA oligos and APE

**Figure 6.**
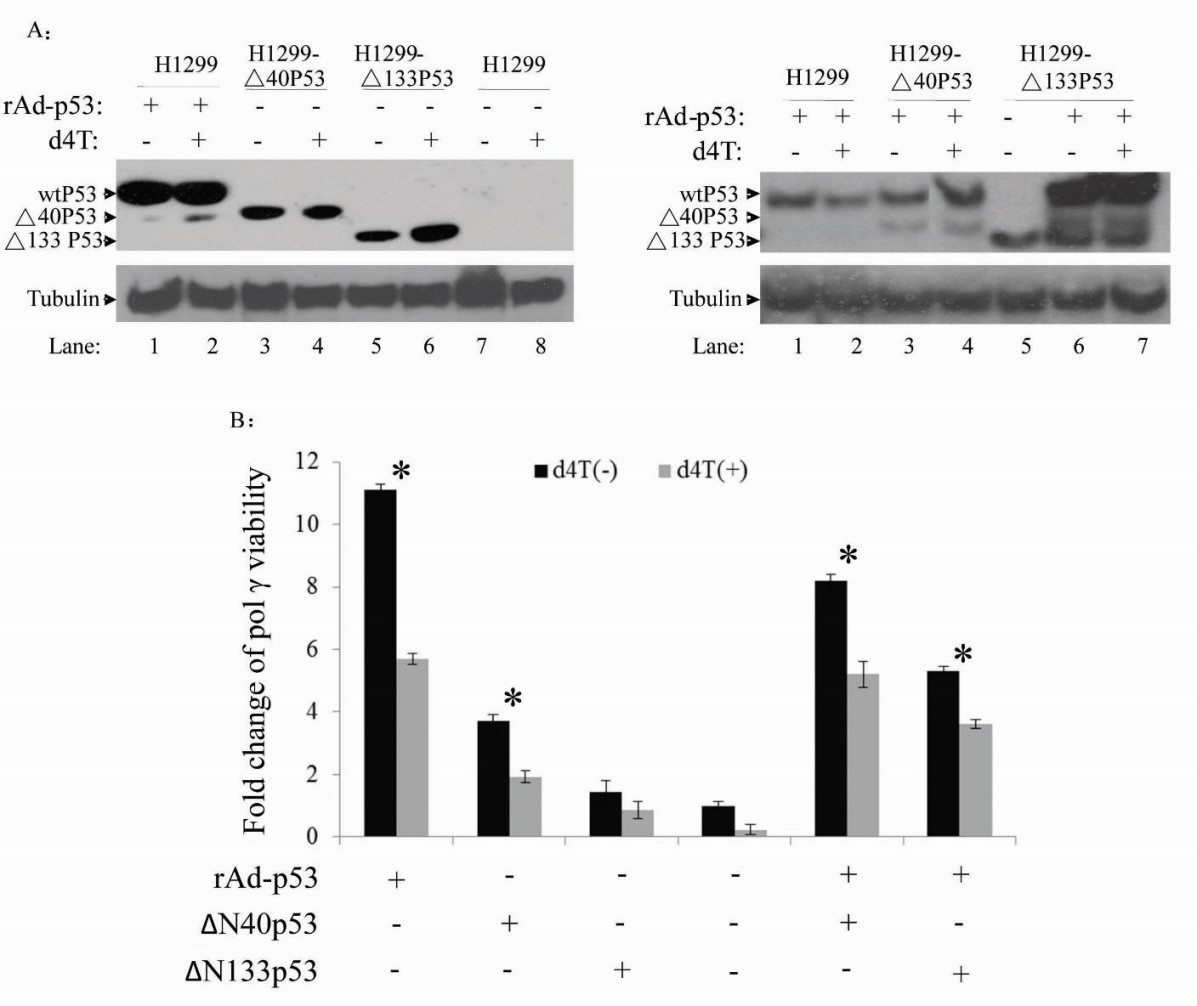
**The high viability of DNA pol γ in mitochondria overexpressing wtp53 and d4T attenuates the activity of mtDNA pol γ**. (A) wtp53, Δ40p53 and Δ133p53 protein expressions under various conditions, detected by Western blotting. (B) The activity of mtDNA pol γ in mitochondria isolated from the cells with various p53 isoforms. (C). Efficacy of p53 and its isoforms on the activity of DNA pol γ in the presence and absence of d4T. Data represent the average of three independent experiments + SD, *P<0.05.

The results in Figures 2-4 show that Δ40p53 and Δ133p53 each have a significant negative effect on wtp53-induced mtDNA pol γ activity. To analyze the relationship of these isoforms with mtDNA damage, we constructed ^32^P-labeled oligonucleotides containing one AP site as a template (AP-oligonucleotide). APE (100 ng) was then added to the reaction mixture and the binding ability of p53 to the AP-oligonucleotide was examined by gel shift mobility assay. The results show that Δ133p53 alone cannot interact with the AP-oligonucleotide and that Δ40p53 has only a weak binding capacity. APE alone can interact with the AP-oligonucleotide and this binding is enhanced following an increase of mitochondrial protein concentration within the reaction system (Fig. 5A). Although Δ133p53 alone cannot interact with the AP-oligonucleotide, it can however, significantly inhibit APE binding to the AP-oligonucleotide (Fig. 5A). Also, wtp53 can not only interact with the APE-AP-oligonucleotide but can also significantly enhance the interaction of APE with the AP-oligonucleotide (Fig. 5B, lanes 10-12). Furthermore, we determined whether d4T can affect the interaction of APE with the AP-oligonucleotide or the p53-APE-AP-oligonucleotide. The results show that d4T does not affect the APE-AP-oligonucleotide interaction or the interaction of p53 with APE. The Co-IP with anti-p53 polyclone antibody further confirmed that only wtP53 interaction with APE 1 and pol γ activity can be stimulated by endogenous wtp53 and Δ40p53 but not by Δ133p53 (Fig. 5C); wtp53 plays a significant role in maintaining mitochondrial genetic stability [21, 22]. However, whether endogenous p53 isoforms alone or with wtp53 can affect DNA pol γ activity is still unclear. We further examined *in vitro* DNA pol γ activity using our previously published technique [41]. In order to study the association of p53 isoforms and DNA pol γ activity, we first isolated whole mitochondrial extracts and identified the expressions of wtP53, Δ40p53 and Δ133p53 (Fig. 6A). The results show that d4T did not affect the expression of wtP53 or Δ40p53, but led to an increase in Δ133p53 levels (Fig. 6A). The mechanism by which this occurs is still unclear.

We then identified the contribution of wtp53, Δ40p53 and Δ33p53 to pol γ activity in d4T free and d4T treated cells (Fig. 6B). The results show that the DNA pol γ activity in the rAD-p53 infected H1299 cells is 5.5 fold higher than that in the uninfected H1299 cells. The DNA pol γ activity in the Δ40p53-H1299 cells was 2.5 fold higher than that from the H1299 cells, but was significantly lower than that from rAD-p53 infected H1299 cells. These results suggest that the N-terminal 40 amino acids of p53 play a critical role in stimulating the activity of DNA pol γ. However, the Δ133p53 isoform in Δ133p53-H1299 cells had no effect on the activity of DNA pol γ. When Δ40p53-H1299 was infected with rAD-p53, the mtDNA repair capability was similar to rAD-p53 infected cells alone. This suggests that Δ40p53 does not affect the function of wtp53 in mtDNA repair. Following the transfection of Δ133p53-H1299 cells with rAD-p53, the mtDNA repair capability was significantly lower than that in rAD-p53 transfected H1299 cells alone, indicating that Δ133p53 plays a negative role in p53 induced mtDNA repair. When cells were pre-treated with d4T for 6 hours, the DNA pol γ activity was significantly lower than in untreated cells. The DNA pol γ activity in H1299, Δ40p53-H1299, Δ133p53-H1299 and rAD-p53 transfected H1299 cells following treatment with d4T was reduced to 20% (about 45% of the original levels) compared with untreated cells. These findings suggest that Δ40p53 and Δ133p53 alone or co-expressed with wtp53 have different roles in the activation of mtDNA pol γ.

## DISCUSSION

Various therapy-limiting adverse effects observed in HIV-infected patients treated with NRTIs are linked to altered mtDNA replication and subsequent disruption of cellular processes [6, 42]. The mitochondrial toxicity of NRTIs is due to the inhibition of mitochondrial DNA pol γ, resulting in a blockade of mtDNA replication. Two important factors affecting the stability of mtDNA are that mtDNA exists in a high ROS environment and that DNA pol γ is the only polymerase responsible for the repair of damaged mtDNA [7, 43]. Previous studies have shown that p53 plays a direct role in maintaining mtDNA genomic stability through interaction with DNA pol γ or with mitochondrial transcription factor A (mtTFA) and that it also plays an indirect role via up-regulation of p53R2 expression [23, 44, 45]. Recently, more than nine p53 isoforms have been reported, which play different roles in DNA repair pathways, cell-cycle checkpoints and cellular apoptosis [27]. However, the role of p53 and its various isoforms in mitochondrial toxicity induced by NRTIs is still unclear. In this study, we identified the role of wtp53 and its isoforms (Δ40p53 and Δ133p53) in mtBER activity following treatment with d4T.

The Δ40p53 protein still contains a part of the p53 transactivation domain and it can activate gene expression after transfection through a second transactivation domain located between amino acids 43 (43aa) and amino acids 63 (63aa). The wtp53 and Δ40p53 together can also strongly stimulate P21 gene transcription [46]. Isoform Δ133p53 may affect cell aging through inhibition of the cell cycle, DNA double-strand break repair and cell senescence [47]. Whether these isomers can enter the mitochondria and affect repair of mtDNA that has been damaged by ROS and by the mitochondrial toxicity of NRTIs remain unclear. Thus, we isolated and purified the mitochondria from transfected Δ40p53-H1299 and Δ133p53-H1299 cells and performed Western blotting assays. The results show that the Δ40p53 and Δ133p53 isomers can enter the mitochondria in both stress and stress-free conditions (Fig. 1). The functional form of p53 is a tetramer. Δ40p53 alone or with wild-type p53 can form dimers and tetramers [35]. In our study, using purified mitochondrial extracts, we show that Δ40p53 and Δ133p53 alone or with wtp53 can also interact to form dimers and tetramers in the mitochondria. Therefore, we speculated that Δ40p53 and Δ133p53 can, directly or indirectly through wtp53, have an impact on mitochondrial function and affect DNA pol γ activity. The *in vitro* studies we performed with the p53 fusion protein show that Δ40p53 alone enhanced the activity of mtDNA pol γ while Δ133p53 itself had no impact, suggesting that the proline rich zone between the core domain and the second transactivation domain in p53 plays an important role in activating DNA pol γ. Then, we confirmed our finding with stable expression of Δ40p53 and Δ133p53 cell lines. Compared with wtp53, endogenous Δ40p53 only weakly enhanced DNA pol γ activity. Although some reports have shown that Δ40p53 combined with wtp53 can stimulate transcription of some target genes, no report has demonstrated how their interaction affects mtDNA pol γ activity. There are also no studies on the effect of Δ133p53 with wild-type p53 on cell function. We found that Δ40p53 and Δ133p53 in the mitochondria can form both dimmers and tetramers with wild-type p53 and inhibit the ability of wtp53 to enhance the activity of DNA pol γ. Δ133p53 can significantly inhibit the activity of DNA pol γ and this is enhanced by wtp53. Δ133p53 and Δ40p53 in normal cells could increase the impact of mitochondrial DNA damage and BER, which may be more meaningful to the mitochondrial toxicity caused by NRTIs.

Stimulation of BER by p53 is correlated with its ability to interact directly with both APE and pol β [29]. Our analysis shows that the Δ133p53 protein cannot interact with the APE-AP-oligo complex and that Δ40p53 has only weak interactions with the APE-AP-oligo complex; only wtp53 in mitochondrial extracts could strongly interact with the APE-AP-oligo complex. It is suggested that the N-terminal of p53 plays an important role in the interaction of p53 with APE. This finding could explain why only wtp53 had mtBER viability but this needs further verification.

The functional form of d4T in cells is d4T-TP. Until now, the role that p53 and its isoforms play what kind of role in d4T-induced mitochondrial toxicity has been unclear. We found that p53 and Δ40p53 can stimulate DNA pol γ activity, but that Δ133p53 could not regulate DNA pol γ activity. Compared with H1299, our studies showed that Δ133p53-H1299 does not have any significant effect on DNA pol γ activity in d4T treated and un-treated cells. Δ133p53 negative regulated p53 in stimulation of pol γ activity at both presence and absence of d4T. The mechanisms need to be further explored.

### Conclusion

Taken together, our data show that Δ133p53 negatively regulates p53’s stimulation of pol γ in the presence and absence of d4T. We believe that our results are benefit for curing AIDS.
